# Rehydration with fructose worsens dehydration-induced renal damage

**DOI:** 10.1186/s12882-018-0963-9

**Published:** 2018-07-13

**Authors:** Tamara Milagres, Fernando E. García-Arroyo, Miguel A. Lanaspa, Gabriela Garcia, Takuji Ishimoto, Ana Andres-Hernando, Masanari Kuwabara, Thomas Jensen, Yuka Sato, Jason Glaser, Laura G. Sánchez-Lozada, Richard J. Johnson, Carlos Roncal-Jimenez

**Affiliations:** 1 Division of Renal Diseases and Hypertension, Nephrology Division, Mail Stop C281, University of Colorado Anschutz Medical Campus, 12700 East 19th Ave 7th Floor Offices, Aurora, CO 80045 USA; 2 Laboratory of Renal Physiopathology, Instituto Nacional de Cardiologia, Ignacio Chavez, Mexico City, Mexico; 3 La Isla Network, Léon, Nicaragua

**Keywords:** Fructose, Mesoamerican nephropathy, Heat stress, Vasopressin

## Abstract

**Background:**

Increasing evidence suggests heat stress induced chronic kidney disease (CKD) may be mediated by endogenous fructose generation and may be exacerbated by rehydration by fructose-containing solutions. We have recently reported a model of CKD induced by heat stress. Here we test the hypothesis that rehydration with fructose may induce worse kidney injury than rehydration with equal amounts of water, and we also test if this fructose-induced injury is associated with activation of inflammasomes in the kidney.

**Methods:**

Mice were recurrently exposed to heat (39.5 C^0^ for 30 min/h, 5 times daily for 5 wks) with rehydration consisting of 6 ml each night of water (Heat, *n* = 7) or fructose (Heat+F, 10%, *n* = 7), and were compared to control mice on water (Control, *n* = 7) or fructose (Fructose, *n* = 7). Various markers of renal injury were assessed.

**Results:**

Compared to control animals, there was a progressive worsening of renal injury (inflammation and fibrosis) with fructose alone, heat stress alone, and heat stress with fructose rehydration (*P* < 0.01 by ANOVA). The combination of heat stress with rehydration with fructose was associated with increased intrarenal expression of the inflammasome markers, NLRP3 and IL-18, compared to heat stress alone. In addition, heat stress with or without fructose was associated with increased expression of caspase − 3 and monocyte chemoattractant protein-1 levels. Fructose administration was also associated with an increase in serum copeptin levels (a biomarker of vasopressin) and elevated copeptin was also observed in mice undergoing heat stress alone.

**Conclusions:**

These studies suggest that heat stress may activate intrarenal inflammasomes leading to inflammation and renal injury, and provide evidence that rehydration with fructose may accelerate the renal injury and inflammatory response.

## Background

Heat stroke is a concern throughout the world, not only as a complication of exercise (such as marathon running) and of military campaigns, but also for individuals working outside in both rural settings and in hot urban environments [1–6]. Epidemics of heat stroke associated with extreme heat events (heat waves) were reported in Chicago in 1995 and Europe in 2003 [1, 7–10]. Indeed, heat stroke is likely to become more common as temperatures rise, for now nearly 75% of heat extremes can be attributed to climate change [11, 12].

Most emphasis on heat stroke relates to the acute risk for multi-organ failure and death. However, heat stroke has also been associated with residual liver and kidney damage [13, 14]. More recently, we have raised the hypothesis that chronic recurrent heat stress might be able to induce chronic kidney disease (CKD), and we have hypothesized this may be of relevance to the epidemics of CKD being observed in among agricultural workers in various hot communities in Central America, India, Sri Lanka and elsewhere [15–17].

To investigate this hypothesis, we developed a model of CKD induced in mice by repetitive heat stress and dehydration over a 5 week period. Using this model, we found that if mice were hydrated during the heat stress period, they did not develop renal injury, whereas if they hydrated after the heat/dehydration period, that they developed mild tubulointerstitial kidney disease [18]. In this same study we found evidence that heat stress/dehydration caused activation of aldose reductase in the kidney, an osmotically-sensitive enzyme that converts glucose to sorbitol, which is then further metabolized in the renal cortex to fructose. Fructose is known to be metabolized in the proximal tubule by the enzyme fructokinase, and this results in a drop in ATP levels, the generation of uric acid, and a burst of oxidative stress that can cause tubular injury [19]. Indeed, mice lacking fructokinase were protected from the renal injury of recurrent heat stress [18].

Given that many individuals working in the rural communities hydrate themselves with sugary beverages, including soft drinks and fruit juices, one might hypothesize that rehydration with fructose-containing solutions might exacerbate our model of renal injury of heat stress and dehydration. In this regard, recently Garcia-Arroyo et al. developed a model of mild heat stress and dehydration in rats in which rats were exposed for only 1 h a day to heat (36 °C) followed by 2 h rehydration with water or a soft drink mixture containing 11% fructose-glucose mixture [20]. This mild model of dehydration does not result in chronic interstitial fibrosis but does manifest with tubular injury and intrarenal oxidative stress [20]. Importantly, this study found that rehydration with a sugary beverage resulted in worse tubular injury and oxidative stress.

We therefore tested the hypothesis that fructose might accelerate heat stress induced CKD in our model. Furthermore, recently it has been recognized that subjects developing CKD in Central America often have evidence of intermittent acute renal injury with a predominance of inflammatory cells on their biopsy [17, 21]. This was viewed as counter to the generally held idea that the acute renal injury might be more similar to acute tubular necrosis. However, heat stroke is also associated with a pronounced renal inflammatory infiltrate [17]. We thus investigated if heat stress might be associated with an increase in inflammasome-associated proteins, and tested the hypothesis that this might be worsened by fructose ingestion.

## Methods

### Animals

Male 8-week old C57BL/6 J wild type mice (WT, Jackson Lab, Bar Harbor, ME) were maintained in temperature- and humidity-controlled specific pathogen-free conditions on a 14-h dark/10-h light cycle and fed regular diet ad libitum (Harlan Teklad; no. 2918, containing 58% carbohydrate, 24% protein, and 18% fat), with free access to tap water.

### Experimental design

The experimental study consisted of four groups (*n* = 7 each): WT control (**Control**); WT fructose (**Fructose**); WT mice exposed to heat and water restriction with water rehydration (**Heat**); and WT mice exposed to heat and water restriction with fructose-containing drinking water (10% fructose) as the rehydration fluid (**Heat + F**). The heat stress-dehydration protocol has been previously reported and consisted of placement of mice in a heat chamber set at 39.5° Celsius for 30 min per hour over 8 h (7 episodes/day), 5 days/week, for 5 weeks [18]. In between heat periods mice were allowed to rest at room temperature. Fluids were restricted during the heat/dehydration period, and then 6 ml of rehydration fluid per day were provided per mouse afterwards with each mouse housed in its own cage using millimetric water bottles, consisting of water in the **Heat** group and fructose water in the **Heat + F** group. This degree of rehydration fluid was calculated based on the overall 24 h fluid intake required by mice of this age and weight in our past study [18]. Mice were sacrificed two hours after the last cycle of heat stress (prior to rehydration) at the end of the 5th week by anesthesia (isoflurane) with cardiac exsanguination and collection of serum, puncture of the bladder for collection of urine, and removal of kidney tissues for analyses.

All experiment were conducted with adherence to the National Institute of Health Guide for the Care and Use of Laboratory Animals. The animal protocol was approved by the Animal Care and Use Committee of the University of Colorado.

### Biochemical analyses

Urine was collected at the end of the study from the bladder and were analyzed for urine osmolality using the Freezing-Point Osmometry method (Advance Instruments Micro Osmometer- Norwood Massachusetts USA). Serum and urine creatinine concentrations were analyzed with the high-performance liquid chromatography–tandem mass spectrometry method [22]. Urinary albumin was measured using a Colorimetric Albuwell Assay Kit (Exocell Co., PA). Serum copeptin levels were measured using Elisa enzyme- linked immunosorbent Assay kit for copeptin (Cloud-Clone Corp., Houston TX) Serum fructose was measured using the EnzyChrom Fructose Assay Kit (Bioassay Systems, Hayward, CA) and serum uric acid was measured using QuantiChrom Uric Acid assay kit (BioAssay Systems).

Kidney tissue samples were homogenized in a buffer containing 2 mM MgCl_2_, 1 mM EGTA, 1 mM DTT, and 0.5% (vol/vol) Triton X-100. Homogenates were centrifuged at 13,000 rpm for 10 min (4 °C) and protein in the collected supernatant quantified (BCA protein assay Kit - Pierce, Rockford, IL). Renal fructose and uric acid levels were assessed by utilizing the Bioassay Systems kits (see above) and values normalized to protein lysate concentration. Renal monocyte chemoattractant protein (MCP-1) was measured on cortical lysates by Mouse MCP-1 ELISA Kit (Invitrogen ThermoFisher – Grand Island NY-USA) and corrected for total protein (Pierce, BCA protein assay Rockford, IL-USA).

### Histology

Kidney tissues were fixed in 10% formalin or methyl Carnoy’s and embedded in paraffin. Three micron axial sections of the kidney were stained with periodic acid-Schiff reagent (PAS). Proximal tubular brush border loss was assessed by immunostaining for angiotensin-converting enzyme (ACE) using antimurine ACE antibody; (R&D, Minneapolis, MN). Renal fibrosis was determined by immunohistochemical staining for type III collagen with a goat anti-type III collagen antibody (Southern Biotech, Birmingham-AL-USA). Macrophage infiltration were detected using Rat Anti-Mouse F4/80 antibody (Serotec, Oxford, UK). The number of positive cells for F4/80 was counted using an Aperio scanner (Aperio Technologies, Vista, CA). Additionally, we evaluated the inflammatory response by immunostaining for Nod-like receptor family-3 (NLPR3) with rabbit anti-mouse NRLRP3 (Novus Biologicals, Littleton CO) and interleukin 18 (IL-18) using rabbit polyclonal anti-IL-18 antibody (Abcam, Cambridge MA). Caspase-3, involved in apoptosis, as detected using a rabbit polyclonal anti-caspase 3 antibody (Abcam). The software allows color recognition and positive cells were identified as % positive color saturation at 20 magnification in a blinded manner using at least 15 fields for each biopsy sample. For the fibrosis and ACE staining, digital images at 20X magnification of approximately 10 cortical fields were analyzed using Image scope of Aperio Scanner software. The percent positive area was determined as the 3,3-diaminobenzidine-positive pixel values per negative pixel values in each section.

### Validation study

We also determined whether inflammasomes and apoptosis-related pathways might be stimulated in the kidney in another model of heat stress and whether this response would be amplified by rehydration with fructose-containing drinks versus water. Specifically, we obtained tissues from male Wistar rats that had been exposed daily (5 times/week for 4 weeks) to one hour of heat stress (37 °C for one hour) followed by two hours ad libitum hydration with either water or a 10% fructose solution. The rest of the day the rats had regular water and normal rat chow ad libitum. This model has previously been shown to result in low grade tubular injury and renal oxidative stress that was worse in the fructose-rehydrated rats [23, 24]. We used renal tissues from one of these studies [24] to evaluate for the presence of NLRP3, caspase-3 and IL-1β by western blot [24] using specific primary antibodies (anti-NLRP3 antibody (ab4207, Abcam), anti-Cleaved Caspase 3 (Asp 175) antibody (Cell Signaling), and anti-IL-1β (C-20, Santa Cruz Biotechnology), with loading controlled using an anti-beta actin antibody (Genetex).

### Statistical analysis

Statistical analyses were performed using the GraphPad Prism version 6 (GraphPad Software, Inc. La Jolla, CA). All data are presented as the mean ± s.e.m. Independent replicates for each data point (n) are identified in figure legends and one-way analysis of variance (ANOVA) with the Bonferroni post hoc test used for individual comparisons. We also used the Student’s t-test to specifically compare heat alone (**Heat**) to heat plus fructose (**Heat + F**) groups since the experiment was designed specifically for this comparison (see comments in limitation section). *P* < 0.05 was regarded as statistically significant.

## Results

### General findings

Recurrent heat stress and dehydration were induced in two groups of mice over a 5 week period in which the only difference was that one group received water rehydration (Heat) and the other group received the same amount of drinking water containing fructose (10%) (Heat+F). We also studied two groups of mice not exposed to heat stress/dehydration administered the same dose of water or fructose-containing water. While each day the mice undergoing heat stress and dehydration would lose significant weight (14.2±0.8 vs 13.8±1.2% in the Heat vs Heat+F groups, p = NS), they were able to fully rehydrate at night and no mortality was observed. At the end of the study there were significant differences in weight by one way ANOVA, with the fructose control showing higher weight compared with the heat stress/dehydration group (*p* = 0.003) and heat stress/dehydration groups plus fructose group (*p* < 0.001), but with no differences between the other two groups (Table 1).

**Table 1:** General Characteristics of Experimental Groups

Parameters	Control	Fructose	Heat	Heat+F	Anova *p* values
Body weight Basal (g)	29.5 ± 2.1	29.3 ± 2.0	30.0 ± 2.3	30.2 ± 2.1	NS
Body weight After 5 W (g)	31.3 ± 3.6	33.3 ± 2.1	28.8 ± 1.9	28.3 ± 1.2	< 0.001
Serum Osmolality (mOsm/Kg)	307 ± 6.1	312.2 ± 6	343.6 ± 16.8	340 ± 29.8	< 0.0001
Serum Copeptin (pg/ml)	10 ± 3.9	25.3 ± 1.4	18.4 ± 4.5	25 ± 4.2	< 0.0001

Comparisons: The Fructose control group has a higher body weight at the end of the study compared to HEAT (*p* < 0.01) and HEAT+F groups (*p* < 0.001) by Bonferroni test. Controls have slower serum osmolality than HEAT (*p* < 0.001) and HEAT+F groups (*p* < 0.01) by Bonferroni. The Fructose group has lower serum osmolality than HEAT (*p* < 0.05). The Controls have lower serum copeptin than Fructose (*p* < 0.001), HEAT (*p* < 0.01) and HEAT+F groups (*p* < 0.001) by Bonferroni test. The Fructose group has higher copeptin levels than HEAT (*p* < 0.05). The HEAT group tends to have lower copeptin levels than the HEAT+F group (*p* = 0.08) by Bonferroni, and is significant by t-test (*p* = 0.049)

### Effect on dehydration markers

Both groups of mice exposed to heat stress and dehydration showed increases in serum osmolarity, and serum vasopressin (determined by measuring serum copeptin) levels (Table 1). Serum copeptin levels increased more in the fructose rehydrated control group compared to the water rehydrated group (Table 1), consistent with studies suggesting fructose can stimulate vasopressin release [25, 26]. The effect of fructose to increase serum copeptin was also greater in the Heat+F compared to the Heat alone group when analyzed by Student’s t-test (*p* = 0.049).

### Effect on renal function

Serum creatinine was elevated in both heat stress groups compared to the control and fructose alone groups (Table 2). Also, the groups exposed to heat stress and dehydration plus fructose had a tendency for elevated albuminuria compared to the control group (*P* = 0.055) or fructose group (*P* = 0.051) by Bonferroni post hoc test. However, no significant differences were noted between the heat stress and heat stress plus fructose group.

**Table 2:** Effect of Fructose with or without Heat on Renal Disease

Parameters	Control	Fructose	Heat	Heat+F	Anova *p* values
Serum Creatinine (μg/ml)	0.38 ± 0.1	0.36 ± 0.1	0.69 ± 0.2	0.72 ± 0.2	*P* < 0.001
Urine Albumin (μg/mg of Cr)	26.1 ± 5.1	27.5 ± 7.5	54.3 ± 28.1	65.5 ± 32.3	*P* < 0.05
ACE Positivity % (proximal tubular brush border integrity)	10.3 ± 2.3	6.6 ± 1.8	6.9 ± 1.6	4.5 ± 3.1	*P* < 0.0001
Monocyte Accumulation (F4/80) %	0.13 ± 0.3	0.13 ± 0.2	0.25 ± 0.3	0.55 ± 0.6	*P* < 0.05
Interstitial Fibrosis (Coll-III) %	0.23 ± 0.1	0.28 ± 0.3	0.43 ± 0.3	0.53 ± 0.4	*P* < 0.0001

Comparisons: The serum creatinine of Control is lower than HEAT (*P* < 0.05) and HEAT+F group (*P* < 0.05) by Bonferroni. Fructose has lower serum creatinine than HEAT (*P* < 0.05) and HEAT+F (*P* < 0.05). Controls tends to have lower urinary albumin than HEAT+F (*P* = 0.055) and Fructose tends to be lower than HEAT+F (*P* = 0.051) by Bonferroni. Controls have more proximal tubular brush border (ACE positivity) than Fructose (*P* < 0.001), Heat (*P* < 0.001) and HEAT+F (*P* < 0.001) by Bonferroni. The Fructose group has more brush border (ACE positivity) than HEAT+F (*P* < 0.01). The HEAT group has more ACE positivity than HEAT+F group by t test (*P* < 0.001). Controls have significantly less interstitial macrophage accumulation than HEAT+F (*P* < 0.05) by Bonferroni. Controls have significantly less interstitial fibrosis than HEAT (*P* < 0.01) and HEAT+F (*P* < 0.001) by Bonferroni post hoc test

### Effect on renal histology

**Fig. 1:**
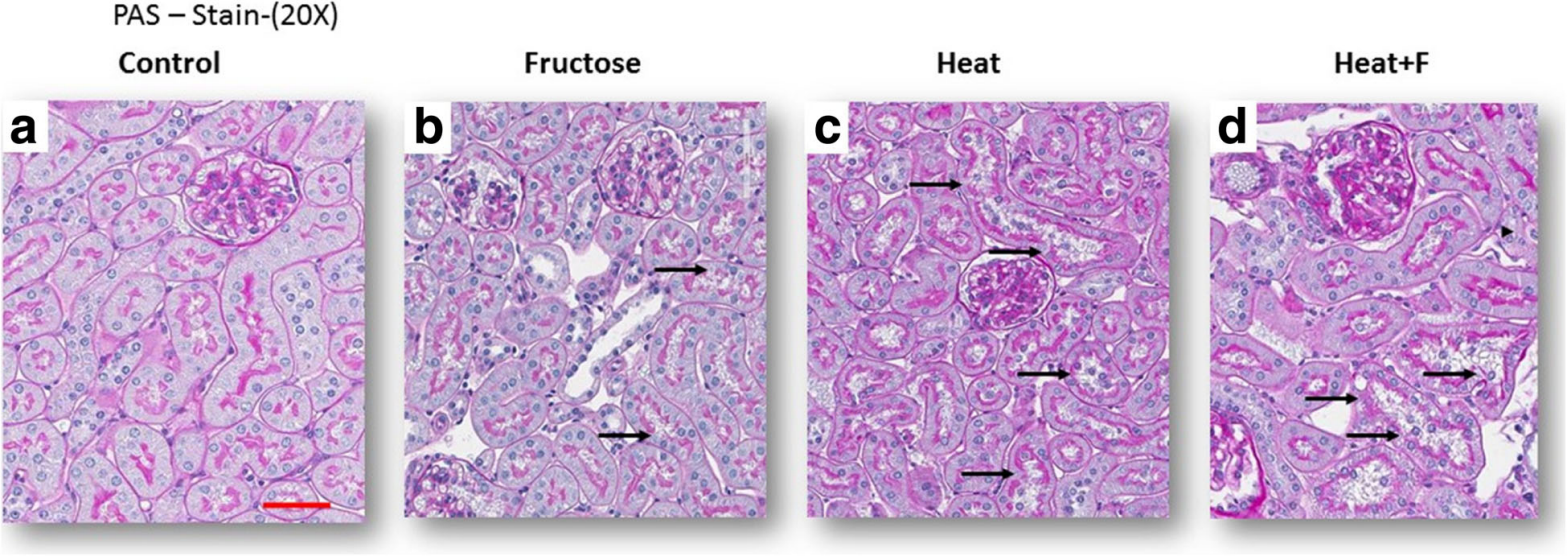
Tubular Damage. Proximal tubular injury, consisting of loss of brush border with tubular dilation (black arrows in PAS stain, 200) is observed in fructose-treated control (**b**), and is worse in mice exposed to heat stress/dehydration with water rehydration (**c**) or fructose (**d**), comparing with normal control group (**a**) (PAS, 200)

**Fig. 2:**
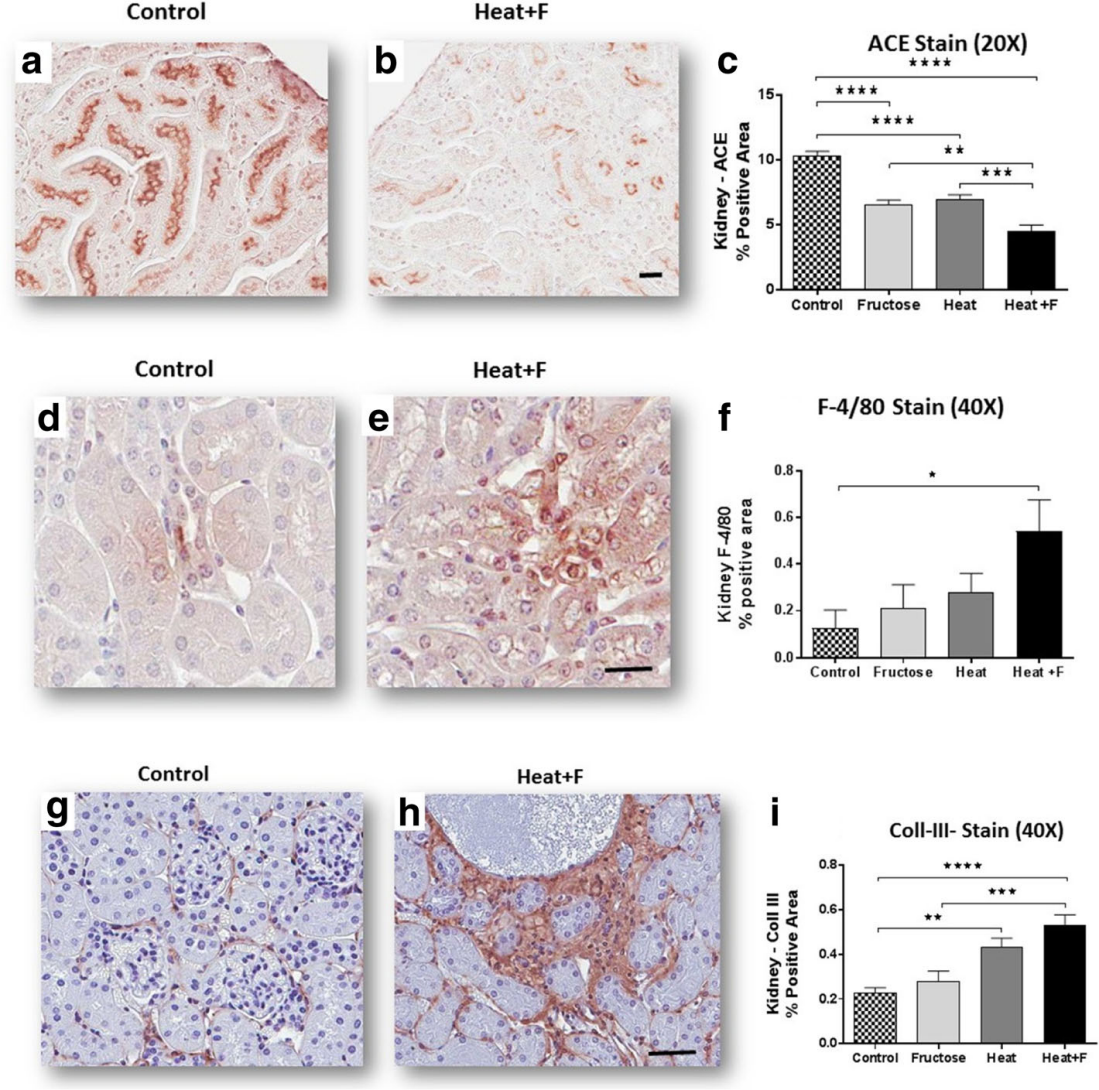
Renal injury. Proximal tubular injury was assessed by staining for proximal tubular brush border for ACE (**a**-**c**, 200), interstitial inflammation was determined by staining for F4/80 positive macrophages (**d**-**f**, 400×), and interstitial fibrosis by staining for type III collagen (**g**-**i**, 200). Heat stress was associated with loss of proximal tubular brush border, interstitial inflammation and interstitial fibrosis which tended to be worse with fructose rehydration. Scale bar 50 μm. Statistical analysis was performed using ANOVA with Bonferroni correction for individual comparisons. Key: *, *p* < 0.05; **, *p* < 0.01; ***p < 0.001, ****, *p* < 0.0001

Compared to controls animals, mice exposed to heat stress and dehydration (HEAT) and (HEAT+F) showed evidence for focal proximal tubular injury (Fig. 1) with loss of proximal tubular brush border as noted by immunostaining for angiotensin converting enzyme (ACE) (Fig. 2a-c, Table 2). The mice receiving fructose as rehydration (HEAT+F) showed more severe loss of proximal tubule brush border compared to heat alone (HEAT) (*P* < 0.001) (Table 2). This was associated with interstitial macrophage infiltration (F4/80 positive cells) (Fig. 2d-f, Table 2). Interstitial fibrosis (noted by collagen III staining) was also increased in the renal cortex of heat stressed mice, but there was no difference between HEAT and HEAT+F groups (Table 2, Fig. 2g-i).

### Effect on sorbitol, fructose and uric acid levels in the renal cortex

**Fig. 3:**
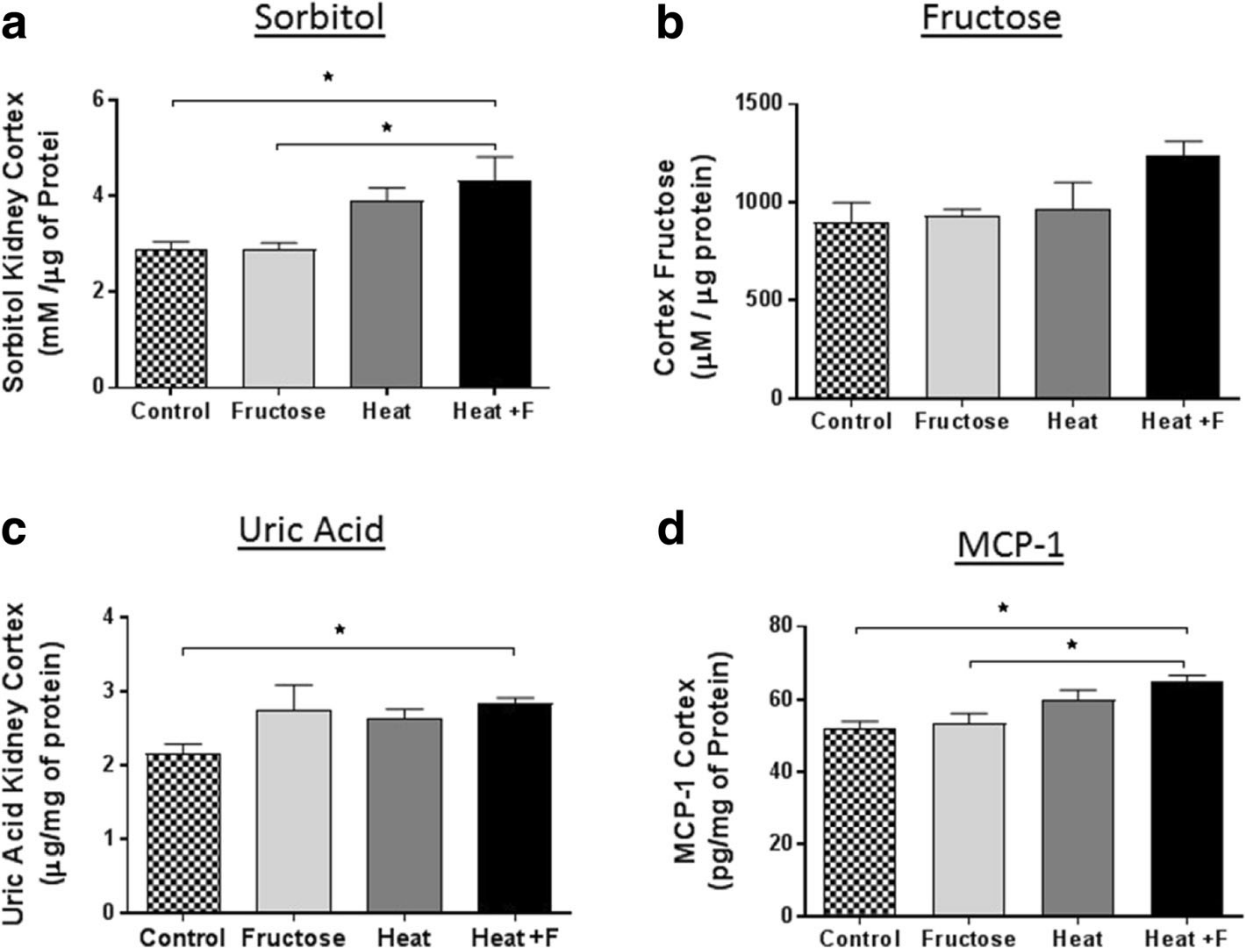
Renal Cortex Sorbitol, Fructose and Uric Acid Levels. **a** Sorbitol levels were increased in heat plus fructose (HEAT+F) exposed mice compared to wild type controls by Bonferroni post hoc test (*p* = 0.013). The Fructose control group also had lower levels of sorbitol than the HEAT+F group (*p* = 0.015). **b** There was no significant differences of fructose in renal cortex for individual group comparisons by Bonferroni post hoc test. **c** HEAT+F group had higher uric acid in renal cortex compared to the control by Bonferroni post hoc test (*p* = 0.042). **d** HEAT+F group had higher MCP-1 in renal cortex than control group (*P* = 0.013) and fructose group (*P* = 0.016) by Bonferroni post hoc test. Key: *p < 0.05

The polyol (aldose reductase) pathway has been reported to be induced in the renal cortex of this heat-stressed model in mice [18]. Sorbitol, the product of aldose reductase, was elevated in both groups of mice exposed to heat stress and tended to be higher in the fructose treated group (HEAT+F) compared to heat stress alone (HEAT), although this was not significant (Fig. 3a). We also measured fructose levels in the renal cortex. Surprisingly, fructose levels in renal cortex were not higher in the heat stress group compared to the control group and other groups (Fig. 3b). Fructose is metabolized in the proximal tubule by fructokinase with the generation of uric acid [19]. Uric acid levels were higher in the renal cortex of heat stress and fructose (HEAT+F) group compared to control group (Fig. 3c).

**Fig. 4:**
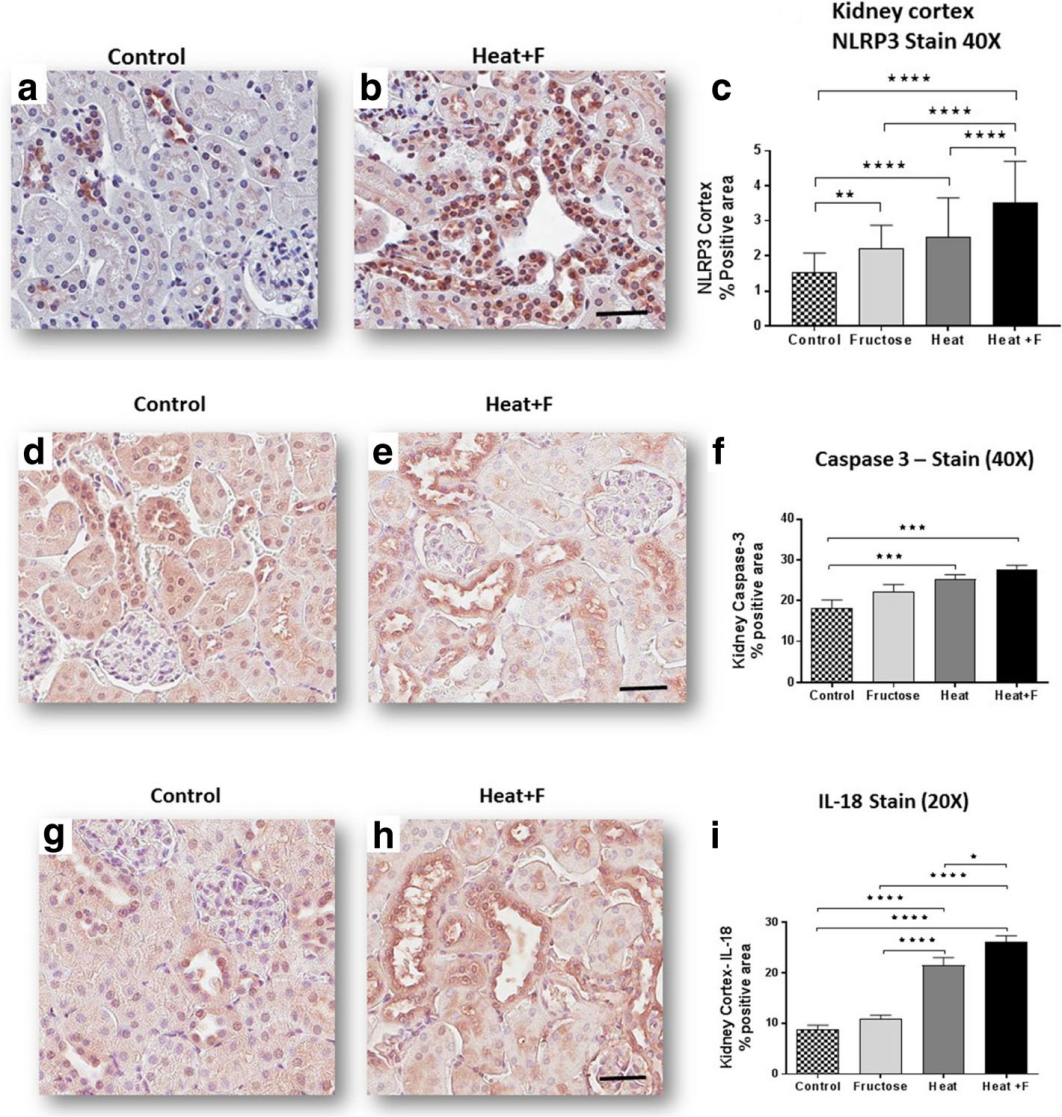
Inflammatory response and renal injury. Renal cortical NLRP3 expression showed stepwise increase with the highest level in the HEAT+F group by quantitation of % NLRP3 positive stain area (**a**-**c**, 400×, Scale bar 50 μm). Caspase 3 expression was higher in heat stress + Fructose (HEAT+F) group compared to the other groups (**d**-**f**, 400× Scale bar 50 μm). Similar findings were shown for Interleukin 18 (**g**-**i**, 400×, Scale bar 50 μm). Key: *, *p* < 0.05; **, *p* < 0.01; ***p < 0.001, ****, *p* < 0.0001

We have previously reported that fructose generated uric acid can stimulate MCP-1 production in proximal tubular cells [19, 27]. Soluble uric acid has also been reported to stimulate inflammasome responses in tubular cells [28]. We therefore measured the renal cortical content of the chemokine, MCP-1, (Fig. 3d) as well as markers of the inflammasome, including NRLP3 (Fig. 4a-c); Caspase 3 (Fig. 4d-f), and the macrophage product, IL-18 (Fig. 4g-i). Specifically, NLPR3, caspase 3 and IL-18, and all were higher in the mice with heat stress that were rehydrated with fructose as opposed to water, with NLRP3 and IL-18 levels were significantly higher in the HEAT+F group compared to HEAT alone.

### Validation study

**Fig. 5:**
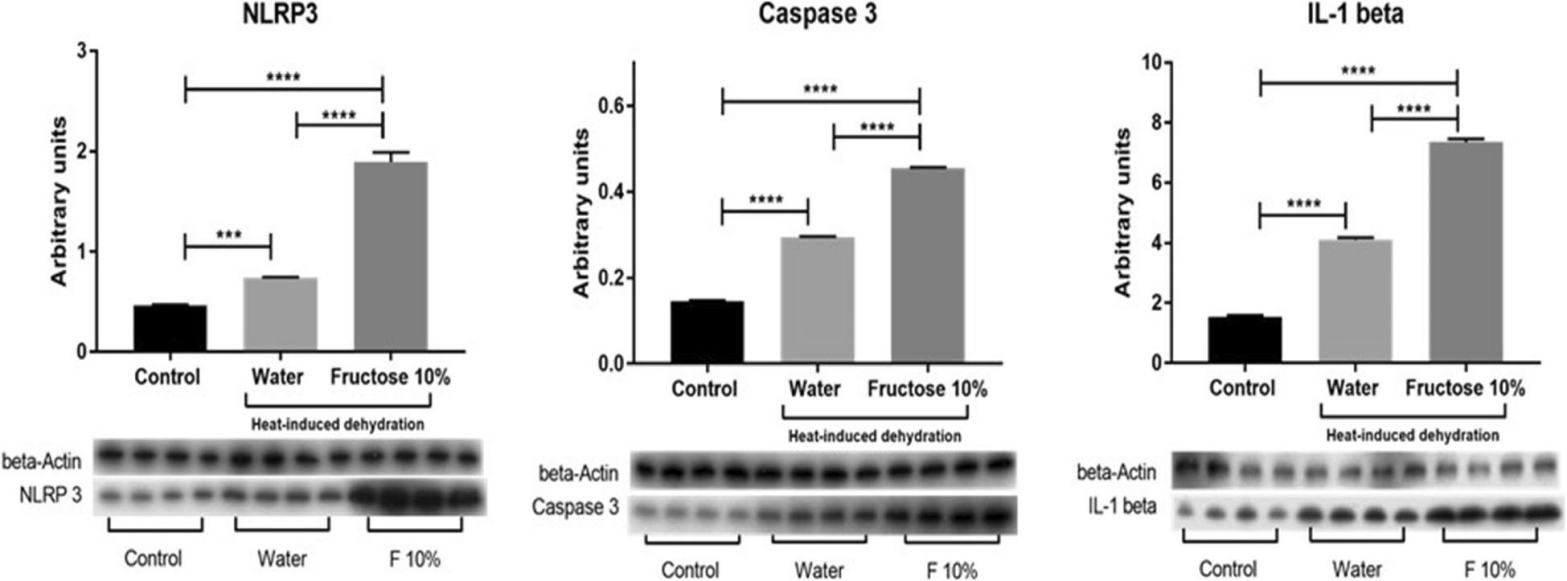
Inflammasome Activation in a Rat Model of Heat Stress. Protein lysates from the kidneys of a rat model of heat stress [24] were used to confirm whether rehydration with fructose containing solutions can increase the expression for inflammasome and apoptosis markers in the kidney. Heat stress with water rehydration could induce an increase in NRLP3, Caspase-3, and IL-1β protein in renal tissues compared to normal controls. However, when rats were rehydrated with fructose (10% in the drinking water), the expression of these inflammasome markers increased markedly and were significantly greater than rehydration with water alone. *N* = 4 per group

We also performed additional analyses on another model of heat stress induced renal injury to determine if fructose rehydration was associated with an induction of inflammasome markers in that model. Specifically, we have reported that Wistar rats exposed to transient heat stress (1 h daily of 37 °C) will develop mild tubular injury and renal oxidative stress that is worse if they are rehydrated following the heat stress with fructose-containing solutions as opposed to water [23, 24]. We obtained some of the rat tissues from one of these prior studies and probed the tissue for markers of inflammasomes and apoptosis by Western blot. As shown in Fig. 5, fructose rehydration was associated with significantly greater expression of the inflammasome markers NLRP3, caspase-3, and interleukin 1β.

## Discussion

Heat Stress and dehydration are well known to cause a ‘prerenal’ type of kidney injury, but it has historically been considered to not be associated with tubular injury and to be fully reversible with hydration. Recently some have proposed that recurrent heat stress and dehydration may be a cause of CKD, based primarily on epidemiological studies performed on sugarcane workers who are developing CKD in Central America [29–32]. To explore this possibility, our group developed a model of CKD in mice induced by repetitive exposure to heat stress over a 5 week period [18]. These mice developed chronic tubulointerstitial inflammation and interstitial fibrosis, both which are characteristic of the CKD in Central America (Mesoamerican Nephropathy) [33].

Here we evaluated the effect of equal hydration with fructose containing water or regular water in our chronic model of heat stress. The primary finding was that fructose worsened the renal tubular injury (as noted by ACE staining) and resulted in marked tubulointerstitial inflammation and a tendency for worse renal fibrosis (although the latter two findings were not significant). In addition, we found evidence for an increase in inflammatory pathways in the renal cortex, including the chemokine MCP-1, inflammasome, and apoptosis markers (NLRP3, caspase 3 and IL-18). Thus, dietary fructose should be viewed as a potential amplifier of the renal injury process, and the mechanism is tightly linked with stimulation of inflammatory processes.

While our model is in mice, we have also developed a model of heat stress associated renal injury in rats [23, 24]. This is a milder model in which rats are exposed to only one hour of heat stress, and rats develop tubular injury without fibrosis associated with renal oxidative stress and systemic inflammation. In this model we have also shown that rehydration with fructose can result in greater renal oxidative stress than hydration with water alone [23, 24]. Since one of the new findings in this study was the finding that fructose might increase the expression of inflammasome markers, we tested kidney tissues from this previously published rat model [24] and confirmed that rehydration with fructose significantly increases the expression of inflammasome and apoptosis markers (NLRP3, caspase 3, and IL-1β) compared to water hydration alone.

The mechanisms by which repetitive heat stress may cause kidney damage has been a ‘hot’ topic. Some studies suggest a role for vasopressin [24, 34], which has long been known to induce some tubular and glomerular injury [35]. Indeed, in the rat model of heat stress associated renal injury, conivaptan, which blocks V1a and V2 receptors, could reduce renal injury associated with recurrent heat stress [24]. We have also reported that stimulation of V2 receptors with desmopressin can accelerate injury in our mouse model [34]. An interaction between fructose and vasopressin is also likely, as we have reported that endogenous fructose (produced in the hypothalamus in dehydration through an osmolality activation of aldose reductase) can stimulate vasopressin synthesis [26], and dietary fructose has also been reported to increase circulating vasopressin levels [23–25]. We also noted an increase in serum copeptin in our fructose hydrated mice. Indeed, mice that cannot metabolize fructose easily (fructokinase knockout) appear to be protected from heat stress induced renal disease [18]. Similarly, conivaptan treatment could block renal injury in the rat model of heat stress, especially in animals rehydrated with fructose [23]. A prime question resulting from this work is whether sports drinks and rehydration solutions that are being used to hydrate individuals in hot environments may be increasing their risk for renal damage.

A weakness in our study was that the renal fibrosis observed in our fructose-rehydrated mice was not significantly worse than heat stress alone, and as such we cannot conclude that fructose definitely worsens heat stress nephropathy. However, fructose hydration was associated with significantly worse tubular injury and expression of inflammatory markers. It is possible that a difference would have been shown if the study had been conducted over a longer period. Nevertheless, the overall trend is suggestive that fructose, and perhaps other sugary beverages, are not likely to benefit and tend to worsen renal injury associated with heat stress and dehydration.

We should state a limitation about statistics. We conducted Bonferroni correction for multiple comparisons. However, since our primary interest was whether there was worse renal injury with heat stress with rehydration with fructose compared to heat stress with water rehydration, we also performed a statistical assessment of these two groups using the t-test. We understand this method is not fair if we accounted for the effect of both heat and fructose. However, we believe that this t-test is acceptable in this case because our aim is to know only the effects of fructose intake under the heat stress condition.

## Conclusion

In conclusion, rehydration with fructose in a model of heat stress and dehydration was associated with significant increased renal inflammation and a tendency for worse renal outcomes compared to equivalent rehydration with water. We recommend additional studies to investigate what concentrations of fructose and glucose may provide benefit for maintaining glucose stores while at the same time minimizing the risk for renal injury in the dehydrated patient.

## Abbreviations

ACE: Angiotensin converting enzyme
ANOVA: One way analysis of variance
CKD: Chronic kidney disease
Heat + F: Heat plus fructose group
IL-18: Interleukin 18
MCP-1: Monocyte chemoattractant protein-1
NLPR3: Nod-like receptor family-3
PAS: Periodic acid-Schiff reagent
WT: Wild type
